# Predicting Patient Mortality for Earlier Palliative Care Identification in Medicare Advantage Plans: Features of a Machine Learning Model

**DOI:** 10.2196/42253

**Published:** 2023-02-20

**Authors:** Anne Bowers, Chelsea Drake, Alexi E Makarkin, Robert Monzyk, Biswajit Maity, Andrew Telle

**Affiliations:** 1 Evernorth Health, Inc St. Louis, MO United States

**Keywords:** palliative, palliative care, machine learning, social determinants, Medicare Advantage, Medicare, predict, algorithm, mortality, older adult

## Abstract

**Background:**

Machine learning (ML) can offer greater precision and sensitivity in predicting Medicare patient end of life and potential need for palliative services compared to provider recommendations alone. However, earlier ML research on older community dwelling Medicare beneficiaries has provided insufficient exploration of key model feature impacts and the role of the social determinants of health.

**Objective:**

This study describes the development of a binary classification ML model predicting 1-year mortality among Medicare Advantage plan members aged ≥65 years (N=318,774) and further examines the top features of the predictive model.

**Methods:**

A light gradient-boosted trees model configuration was selected based on 5-fold cross-validation. The model was trained with 80% of cases (n=255,020) using randomized feature generation periods, with 20% (n=63,754) reserved as a holdout for validation. The final algorithm used 907 feature inputs extracted primarily from claims and administrative data capturing patient diagnoses, service utilization, demographics, and census tract–based social determinants index measures.

**Results:**

The total sample had an actual mortality prevalence of 3.9% in the 2018 outcome period. The final model correctly predicted 44.2% of patient expirations among the top 1% of highest risk members (AUC=0.84; 95% CI 0.83-0.85) versus 24.0% predicted by the model iteration using only age, gender, and select high-risk utilization features (AUC=0.74; 95% CI 0.73-0.74). The most important algorithm features included patient demographics, diagnoses, pharmacy utilization, mean costs, and certain social determinants of health.

**Conclusions:**

The final ML model better predicts Medicare Advantage member end of life using a variety of routinely collected data and supports earlier patient identification for palliative care.

## Introduction

### Background

Approximately 43% of all Medicare beneficiaries are enrolled in Medicare Advantage plans, totaling 24.4 million Americans as of July 2020 [1]. As the Medicare Advantage population lives longer with more chronic conditions, the need for palliative services and serious illness care management becomes increasingly important [2]. Palliative services in Medicare Advantage refer to (nonhospice) primary, specialty, and supportive care services for individuals with serious advanced illness and complex chronic conditions that are typically delivered in the patient’s home or in a clinical outpatient setting. Palliative care not only may provide patients a better quality of life but also can reduce costs by enabling avoidance of unnecessary hospitalizations, diagnostic and treatment interventions, and intensive and emergency department care [3-6].

Although the need for and engagement with palliative care among older adults and Medicare beneficiaries is growing, these valuable services are often underutilized [7-9]. One major cause of lower uptake involves unreliability in provider identification of patients who are appropriate for palliative care. Research shows a clinician’s intuition alone is not the most effective method for recognizing individuals in general practice who could benefit from palliative services [10-12]. Standardized screening tools that rely primarily on diagnostic criteria, medical record information, and patient-reported needs can promote better reliability in clinician identification of palliative patients [13-20]. However, providers and health plans are increasingly leveraging powerful, data-driven machine learning (ML) techniques to help recognize potential candidates for palliative care earlier and more objectively.

### Machine Learning for Palliative Care Identification in Medicare

ML is being adopted across hospital and community-based health care settings as a mechanism to guide early identification of older adults in need of palliative services. ML algorithms attain superior predictive performance from using one or more sources of big data for model training, such as routinely collected medical service claims, electronic medical records, and clinical assessment outcomes [21]. The likelihood of patient mortality within a certain time frame is commonly used as the predictive outcome for ML models intending to identify potential palliative service candidates, because patients who are approaching the end of life are most likely to need and benefit from palliative care [22]. Using ML to identify patients for palliative care not only saves clinicians valuable time but may also improve the efficiency of service delivery to those at highest risk. Early models such as the Charleston Comorbidities Index and Elixhauser score incorporated claims and administrative data to predict mortality of hospitalized older patients [23,24]. Since then, ML models trained using big data from claims and electronic medical records of Medicare beneficiaries (aged ≥65 years) in nonhospital settings have achieved greater predictive performance, with the area under the receiver operating characteristic curve (AUC) values ranging between 0.79 and 0.97 [25-28]. The predictive power of ML for the early identification of palliative care in nonhospitalized Medicare patients can surpass that of clinical screening tools developed for similar purposes [14,16].

Previous research on ML mortality models for earlier palliative care identification in the Medicare population has mainly focused on optimizing and comparing the performance of different model configurations [6,25-29]. That said, evaluating critical features of ML mortality models is also necessary to understand performance variation among different model configurations relative to the patient population, health care setting, and type of data analyzed. Failing to report on the important feature inputs gives inadequate transparency about how the algorithm reached its stated outcomes based on the sources of training data [30]. ML model feature impact reporting appears to be more common in studies analyzing hospitalized Medicare patients [31-33] but has been largely neglected in ML studies that focus on nonhospitalized Medicare beneficiaries [25-28]. Moreover, such prior studies have tapped into various data sources including medical claims, electronic medical records, patient demographics, and clinical assessment information for model training and validation [6,25-29]. The extent to which other, nonmedicalized data are incorporated into these ML mortality models remains unclear, in part due to the lack of discussion around feature impacts. For example, social determinants (eg, socioeconomic status, environmental conditions) are known to influence the mortality and health outcomes of older adults [34,35]. However, previous ML studies in the Medicare population do not clearly indicate if nonmedical data, like measures of the social determinants of health (SDOH), were incorporated as algorithm features [6,25-29,31-33,36].

The important individual features of ML mortality models used to identify palliative care need among nonhospitalized older Medicare patients remain underreported in the current research [25-28]. In an aim to fill this knowledge gap, this study describes the important feature outcomes and performance of a ML algorithm that was developed and validated to predict 1-year mortality of older US adults (aged ≥65 years) enrolled in Medicare Advantage plans. Our predictive binary classification model was routinely supplied with data extracted from medical claims as well as electronic health records (EHRs), patient demographic information, and location-specific index measures of SDOH for purposes of identifying Medicare Advantage plan members who may need to connect to palliative resources. Through this study, we investigated the following objectives:

- To what extent is the performance of a baseline ML model (demographics-based with high-risk indicators) predicting 1-year mortality of Medicare Advantage plan members (aged ≥65 years) improved by adding features capturing patient service utilization, diagnoses, and SDOH?
- What individual features are of top importance in the final ML model iteration?

## Methods

### Model Development

An ML algorithm predicting 1-year mortality among Medicare Advantage plan members was developed by the team at Cigna, a large US commercial health benefits company. The aim was to create a prognostic ML model of mortality risk that could enhance the process of identifying patients for palliative care, with the long-term goal of increasing engagement with community-based, nonhospice palliative services among adults (aged ≥65 years) in Medicare Advantage plans for whom it would be appropriate. Increasing utilization of palliative services can reduce unnecessary high-cost hospital care and improve patient quality of life. An overview of the health plan’s process for identifying and connecting with potential palliative care patients is outlined in Multimedia Appendix 1.

The retrospective data used in the analysis were internally sourced from Cigna’s proprietary administrative records and claims database. These standard data elements are routinely collected to fulfill the operational purposes of the health benefits company; claims and administrative data were only extracted for the purposes of developing the ML algorithm post facto. Security measures for personal health information require all data be completely de-identified by a separate internal team prior to any secondary data analysis to protect member confidentiality. Due to the sensitivity and proprietary nature of the information, data cannot be shared externally.

### Ethical Considerations

Our study methods were in accordance with the ethical guidelines of the 1975 Declaration of Helsinki, and our reporting conforms to the Guidelines for Developing and Reporting Machine Learning Predictive Models in Biomedical Research [37]. The data used in the analysis were retrospective, deidentified, and not originally collected for research nor model development purposes; data were only extracted to develop the ML algorithm after the fact. An internal ethics committee approved and regularly reviewed the project protocol throughout the model development process.

### Sample Inclusion Criteria

Medicare Advantage plan members eligible for inclusion in analysis were all those with continuous health benefits coverage enrollment as of July 1, 2016, through the feature generation period of December 31, 2017, who also had at least one inpatient or outpatient service encounter in their randomly assigned feature generation time frame. Additionally, to be included in the analyzed sample, during the outcomes period (January 1, 2018, through December 31, 2018), patients must have either (1) had continuous enrollment for the 2018 calendar year or (2) became deceased during 2018. This requirement ensured any beneficiaries who disenrolled from their Medicare Advantage plan in 2018 but were not deceased were not counted as patient expirations.

### Machine Learning Method and Training Protocol

Various binary classification ML models were considered. Performance was compared using 5-fold cross-validation. A light gradient-boosted tree model (LightGBM) performed best and was selected based on cross-validation log loss (or cross-entropy loss). The protocol analyzed data from a total sample of 318,774 Medicare Advantage plan members. Features were generated using a training cohort (255,020/318,774, 80% of the sample) with a randomized outcomes time period. Models were further applied to a holdout data set (63,754/318,774, 20% of the sample) to validate and assess generalization to new cases. Data were computed using an instance of DataRobot v6.1.2 (Python 3, custom lightgbm model) running on an on-premise Red Hat Enterprise Linux 7.9 (Maipo) server and with variable resources dedicated via Docker containers (4-8 CPUs each with 32-64 GB RAM).

### Target Outcome

The model’s predicted outcome was defined as any member who expired between January 1, 2018, and December 31, 2018 (1 year). Patients were determined to be deceased based on corresponding plan enrollment data and validation through reporting to the Centers for Medicare and Medicaid Services [38].

### Data Sources and Feature Generation

#### Feature Generation

A SQL script aggregated data to generate predictive features. To determine the date range for model input generation, a randomized cutoff date was assigned to negative and positive cases. We randomized the actual feature generation dates used per customer, so the distribution of start dates was the same for deceased and alive customers. The random date ensured the ML process did not suffer from seasonality and selection bias. Features were built from the 1-year look-back period (ending December 31, 2017) and included 907 unique inputs based on routinely collected data. Data used in model development were information sourced from claims, EHRs, and administrative member records.

#### Claims

Data from claims were primarily used to generate features representing patient service utilization. Diagnosis information was also extracted from claims. Types of claims data included medical service claims, pharmacy claims, and laboratory encounters. Laboratory encounters were based on medical claims for lab-related Current Procedural Terminology (CPT) codes. The actual clinical outcomes (results) of laboratory tests are not part of claims data and were thus not incorporated into the model.

#### Electronic Health Records

Medical data were extracted from EHRs to supplement claims in generating 5 features of high-risk service utilization used in the first iteration of the model (ie, occurrence counts of electrocardiograms, kidney disease, sepsis, ventilator usage, and surgeries). Data from EHRs are aggregated through a third-party vendor partner and are used by the health plan for internal care management and care coordination activities. Not all patients had EHR data on record.

#### Administrative Member Records

Demographic data, as well as information used to calculate measures of SDOH, were extracted from internal administrative member records. Demographic features were patient age (continuous, in years) and gender (male/female). Social determinants index (SDI) scores are a suite of measures in the administrative member record that were developed for internal use. SDI scores are composite measures representing 6 domains of the SDOH: economy, education, language, health, infrastructure, and food access. SDI scores are determined by the member’s census tract, which corresponds to the member’s residential address and zip code [39]. The data associated with the measures in each domain are sourced from public use data such as the US Census and US Department of Agriculture (see Multimedia Appendix 2). Total overall weighted and unweighted SDI scores were also included as features in the model.

### Data Preprocessing

Sample members must have had at least one countable service utilization claim in the randomized feature generation period. No feature observations were removed due to missing data. The data had some categorical fields, such as gender or a categorical indicator of utilization status, but most features were continuous and numeric. Numeric data were not transformed (apart from missing value imputation). Most instances of missing numeric data indicated an individual did not experience a particular type of claim, diagnosis, or event (not due to data quality); such instances were manually coded as 0 to avoid missing values and to represent the patient did not experience the event. Beyond this, DataRobot handles the missing value imputation strategy automatically based on the specified type of imputation algorithm. For the selected model configuration (LightGBM), both continuous/numeric and categorical data had imputed values to represent “missing” data. The final model used ordinal encoding for categorical variables that included a separate category for “missing.” The most common type of missing data was SDI scores, which occurred for 4.9% (15,655/318,774) of the sample population. Age (541/318,774) and gender (647/318,774) data were each missing for 0.2% of the sample.

### Model Training and Validation

Data were split 80/20 into training and holdout partitions, respectively. Within the training partition, additional subdivisions were made to tune parameters and apply early stopping. In a LightGBM tree-based algorithm, early stopping refers to stopping the training process if the model performance does not improve after some consecutive iterations. First, the training data were split (training split 1) to keep 90% for train and 10% for test; this set was used for early stopping. Next, the data were split yet again to create training split 2; using only the training portion of training split 1, we assigned 70% for training and 30% for testing. Training split 2 was used to tune model parameters (ie, num_leaves). After these parameters were tuned, we returned to training split 1 to tune the number of estimators (n_estimators) using early stopping (early_stopping). Key parameters included learning_rate (0.05), n_estimators (550), num_leaves (16), max_depth (no limit), min_child_samples (10), and early_stopping_rounds (200). Both the training and holdout partitions had similar mortality rates of 4% in 2018, indicating the mortality outcome was not biased nor skewed in either the training or validation step.

### Evaluation Measures

Model performance was assessed using AUC, positive predictive value, negative predictive value, true positive rate, true negative rate, average precision, and lift charts focusing on true positives in the top 10% of predictions for the holdout cohort. Based on the data, DataRobot software selected a threshold of 0.16 for comparing the performance metric matrices of the different model iterations. We performed 1-tailed and 2-tailed *z* tests to evaluate significant differences between model iterations with the addition of features. Model performance outcomes for the training data set (255,020/318,774, 80% of the sample) are located in Multimedia Appendix 3. Performance outcomes for the holdout data set (63,754/318,774, 20% of the sample) are presented herein to validate the model and assess generalization to new cases. We report the ranked order importance and absolute (unnormalized) importance values of the top 20 model input features based on Shapley Additive Explanations (SHAP) values [30,40].

## Results

Of the 318,774 patients included in the total sample, 96.1% (306,227/318,774) were determined to be alive, and 3.9% (12,547/318,774) were determined to be deceased during the 2018 outcomes period (see Table 1). Compared with alive patients, deceased patients were older, had higher rates of chronic health conditions (cancer, dementia, stroke, heart failure, and chronic respiratory disease), and had greater average service utilization including emergency room, pharmacy, and laboratory encounters. Deceased patients also had lower SDI scores on average (weighted and unweighted) compared with alive patients.

**Figure 1:**
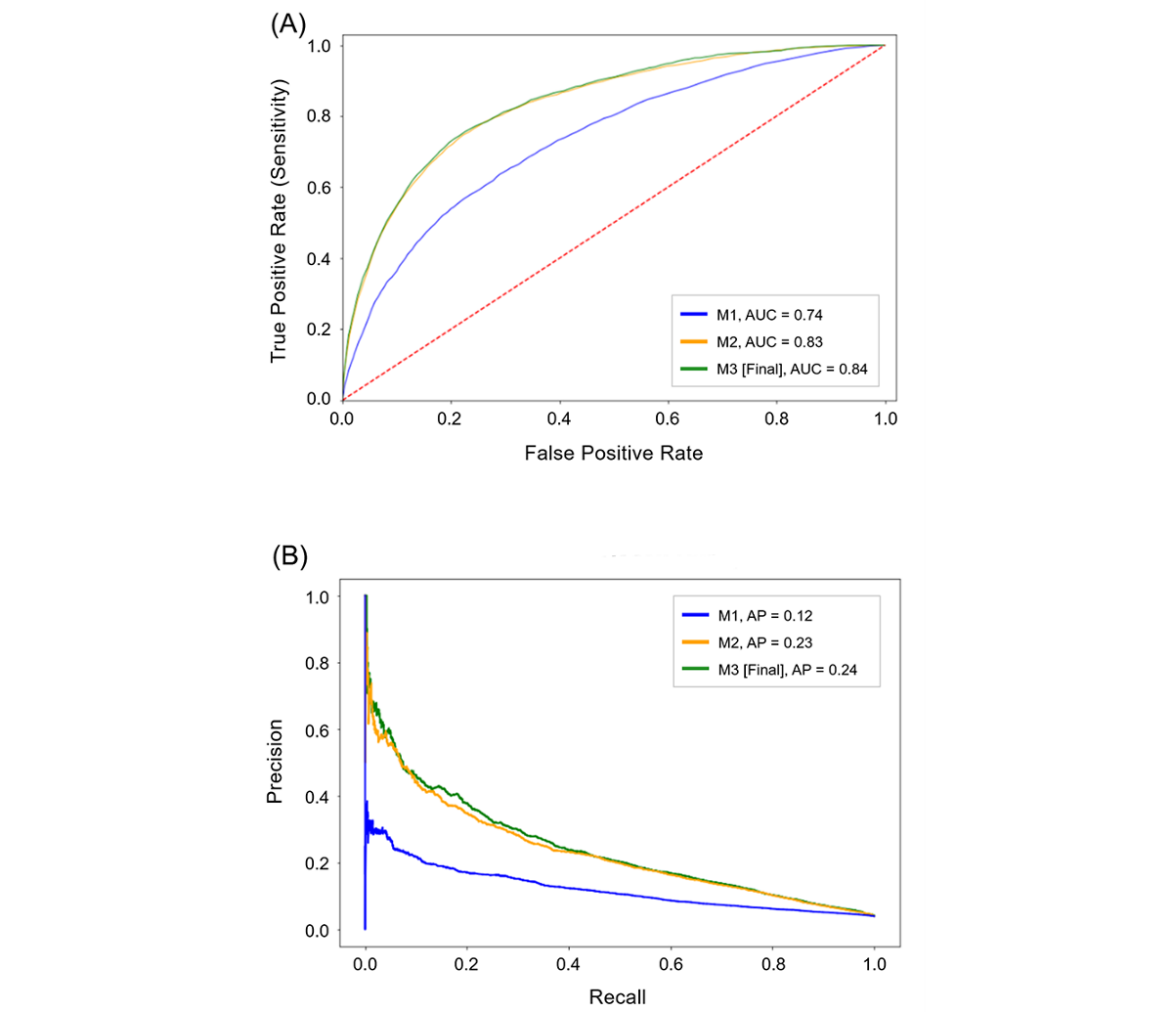
Comparison of Model 1 (M1), Model 2 (M2), and Model 3 (M3) using (A) receiver operating characteristic curves and (B) precision recall curves. AP: average precision; AUC: area under the receiver operating characteristic curve.

**Figure 2:**
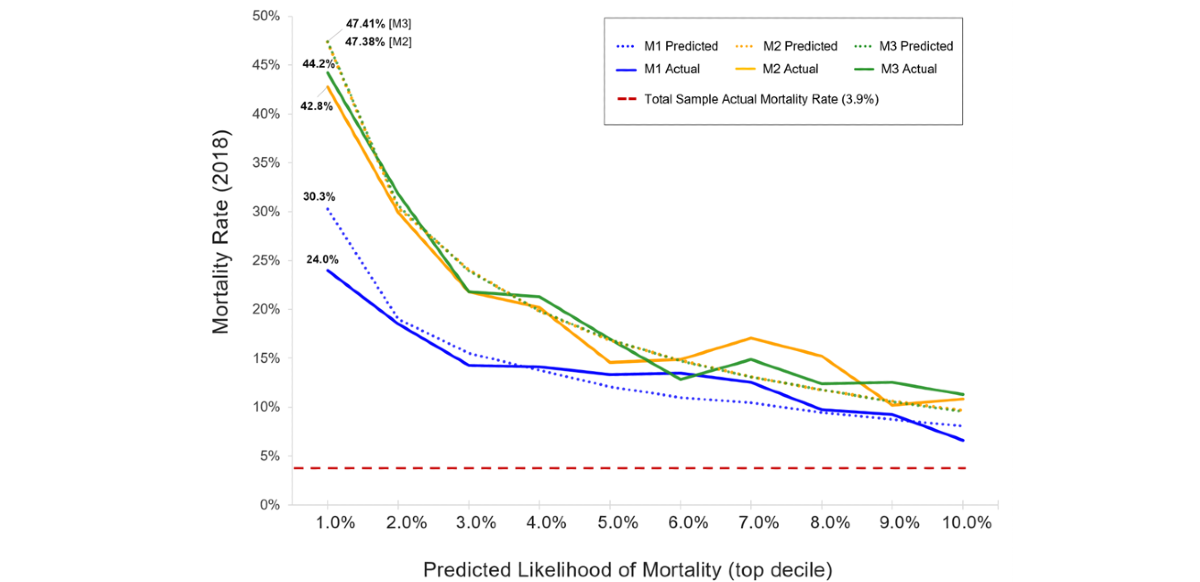
Model mortality outcomes for patients in the top decile of the highest predicted risk. M1: Model 1; M2: Model 2; M3: Model 3.

Table 2 summarizes the ML model development and performance outcomes for the holdout cohort (63,754/318,774, 20% of the sample). The baseline model, Model 1 (M1), included 2 demographic features (age and gender) and 5 features capturing elements of high-risk utilization. Model 1 achieved an AUC value of 0.736 (95% CI 0.728-0.744), which was significantly better than mortality prediction based on random chance alone (*z*=56.4, *P*<.001). In the next stage of development, Model 2 (M2) was created by adding 894 more input features using service claims that captured patient clinical diagnoses as well as individual medical, laboratory, and pharmacy utilization. The M2 iteration had an AUC value of 0.834 (95% CI 0.828-0.840), which was a significant performance improvement compared with M1 (*z*=19.1, *P*<.001). Model 3 (M3), the final model, added 8 features representing SDOH (SDI scores). M3 had the best performance of all the model iterations, with an AUC value of 0.839 (95% CI 0.833-0.845), showing significant improvement over that of M1 (*z*=20.2, *P*<.001). The final model (M3) also has a high degree of specificity in that it accurately predicted patients who were not deceased (negative predictive value=0.971), with the model’s average precision improving with each iteration (from 0.12 to 0.24). Adding the SDI score features to the final model (M3) did not improve the performance of the previous model (M2) to a statistically significant degree (*z*=1.2, *P*=.19); however, there was a significant performance improvement between M2 and M3 in the training cohort outcomes (*z*=0.02, *P*=.02; see Multimedia Appendix 3). Other model performance outcomes of M1, M2, and M3 for the holdout cohort were similar to those of the training cohort (Multimedia Appendix 3), which cross-validates the algorithm. The receiver operating characteristic curves and precision recall curves of the 3 model iterations are charted for comparison in Figure 1. Figure 2 compares the predicted outcomes of M1, M2, and M3 against the actual 2018 mortality rate for those patients in the top decile of predicted mortality likelihood. As features were added with each model iteration, classification of the highest risk members improved. The final model (M3) was superior to both M1 and M2, predicting that those in the top 1% of highest risk would have a mortality rate of 47.4% in 2018 (versus an actual mortality rate of 44.2%).

**Figure 3:**
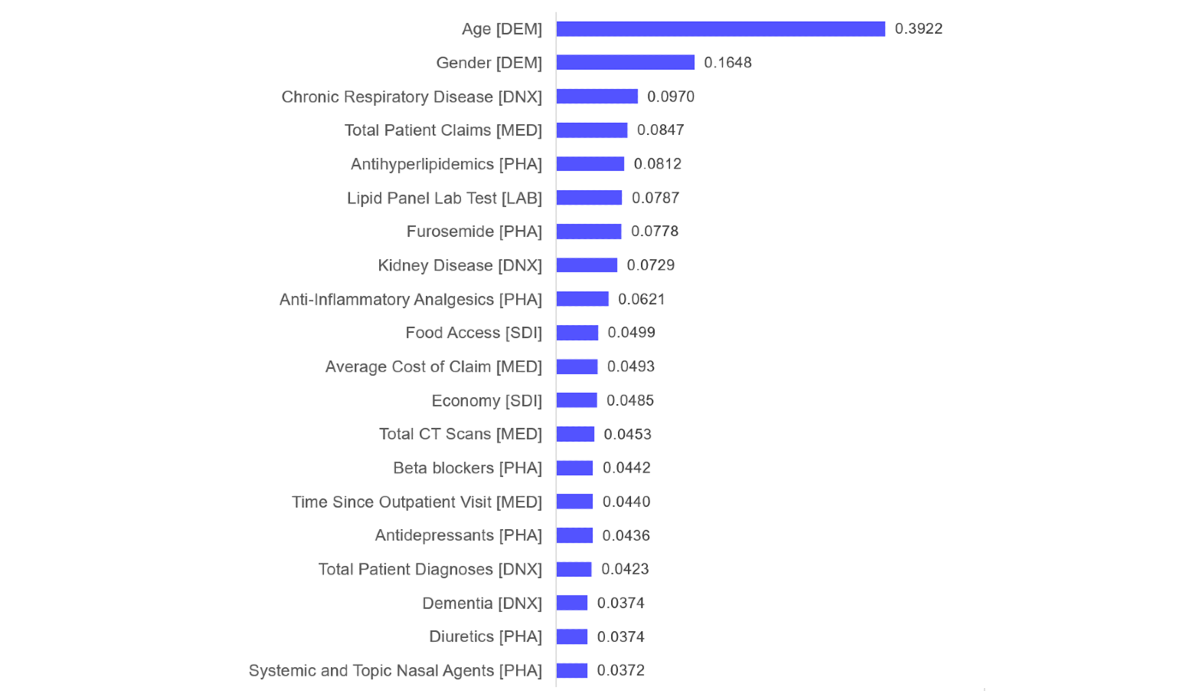
Absolute feature importance in Model 3 (M3). CT: computed tomography; DEM: demographics; DNX: diagnoses; LAB: laboratory utilization; MED: medical utilization; PHA: pharmacy utilization; SDI: social determinants index.

Table 3 reports the top 20 features and their rank among the 907 total inputs of M3. To aid interpretation, features are categorized by demographics, diagnoses, medical utilization, pharmacy utilization, laboratory utilization, and SDOH. The absolute (unnormalized) impact values of the top 20 features are shown in Figure 3. Patient demographics (age and gender) were 2 of the inputs comprising M1, and these were also the most important features contributing to the M3 mortality model. Notably, 3 of the top 20 model features quantify patient information from the total claims data set (total claims, average cost of claim, total diagnoses), and 1 feature was strictly temporal (time since last outpatient visit). Among the top features in M3, 4 inputs captured patient diagnoses, with chronic respiratory disease and kidney disease having the greatest ranked importance (#3 and #8, respectively). Aside from age and gender, kidney disease occurrence was the only other input from M1 to rank in the top 20 features of M3. Additionally, 4 of the 265 medical utilization features were also among the top 20, with total patient claims ranking as the most important in the category (#4) followed by the patient's average cost of claim (#11). Of the 198 pharmacy utilization inputs, 7 ranked in the top 20 features of M3; 3 of these were among the top 10 most important features in the final ML model. These were antihyperlipidemics (#5), furosemide (#7), and anti-inflammatory analgesics (#9). Although there were 201 laboratory utilization inputs, only 1 was among the top 20 most important features in M3 (lipid panel test, #6). The laboratory features were extracted from claims data and only measure utilization; actual results of patient laboratory tests were not a part of the data used to develop the ML model. Finally, 2 of the 8 patient SDI score features ranked among the top 20 features of M3. The important SDOH features predicting mortality in M3 were food access score (#10) and local economy score (#12) based on the plan member's census tract.

**Table 1:** Sample member characteristics.

Characteristic			Total sample (n=318,774)		Alive (n=306,227, 96.1%)		Deceased (n=12,547, 3.9%)
**Gender, n (%)**							
	Female	181,158 (56.8)		174,640 (57.0)		6518 (51.9)	
	Male	136,970 (43.0)		130,941 (42.8)		6029 (48.1)	
	Missing/not available	646 (0.2)		646 (0.2)		0 (0)	
Age (years)*,* mean (SD)			70.7 (11.5)		70.4 (11.5)		77.2 (9.7)
**Medical diagnoses,** **n (%)**							
	Chronic respiratory disease	56,734 (10.4)		52,183 (10.2)		4551 (14.0)	
	Heart failure	54,702 (10.1)		50,254 (9.8)		4448 (13.7)	
	Cancer	44,145 (8.1)		40,985 (8.0)		3160 (9.7)	
	Stroke	21,338 (3.9)		19,327 (3.8)		2011 (6.2)	
	Dementia or Alzheimer disease	15,626 (2.9)		13,018 (2.5)		2608 (8.0)	
	Hypertension	204,405 (37.6)		195,035 (38.2)		9370 (28.8)	
	Diabetes	146,394 (26.9)		139,999 (27.4)		6395 (19.7)	
**Medical service utilization,** **mean (SD)**							
	Total care visits per year^a^	20.8 (39.5)		20.2 (38.2)		36.7 (60.9)	
	Emergency room visits per year	0.4 (1.1)		0.4 (1.1)		0.9 (1.7)	
**Pharmacy utilization,** **mean (SD)**							
	Total unique medications prescribed	9.04 (7.4)		8.9 (7.3)		11.7 (8.3)	
	Number of prescribed medications per day	8.11 (12.0)		8.0 (12.1)		9.8 (9.9)	
**Laboratory utilization, mean (SD)**							
	Total unique lab-related CPT^b^ codes	8.7 (8.4)		8.6 (8.2)		11.7 (11.0)	
**Social determinants index (SDI)^c^, mean (SD)**							
	Weighted SDI score^d^	58.41 (8.65)		58.43 (8.67)		58.09 (8.08)	
	Unweighted SDI score^d^	56.94 (10.12)		56.98 (10.13)		55.91 (9.63)	

^a^Includes all inpatient and outpatient visits.

^b^CPT: Current Procedural Terminology.

^c^Higher is better.

^d^100 points maximum.

**Table 2:** Model summary and performance comparison (holdout cohort).

Measure			Model 1 (M1; baseline)		Model 2 (M2)		Model 3 (M3; final)
Total model features, n			7		899		907
Model input summary			Demographics^a^, High-risk utilization indicators^b,c^		Demographics^a^; High-risk utilization indicators^b,c^; Medical, lab, and pharmacy utilization^c^		Demographics^a^; High-risk utilization indicators^b,c^; Medical, lab, and pharmacy utilization^c^; SDI^d^ scores^a^
**Model performance** **(holdout cohort)**							
	AUC^e^ (95% CI)	0.736 (0.728-0.744)		0.834 (0.828-0.840)		0.839 (0.833-0.845)	
	True positive rate^f^	0.105		0.320		0.2993	
	PPV^f,g^	0.212		0.264		0.2991	
	False positive rate^f^	0.016		0.037		0.029	
	True negative rate^f^	0.984		0.963		0.97126	
	NPV^f,h^	0.964		0.972		0.97129	
	False negative rate^f^	0.890		0.679		0.701	
	AP^i^	0.122		0.233		0.243	
**Performance comparison** **(holdout cohort)**							
	Null hypothesis	AUC_M1_ = 0.5		AUC_M2_ – AUC_M1_ = 0.0		AUC_M3_ – AUC_M2_ = 0.0	
	*z* statistic	56.4		19.1		1.2	
	*P* value	<.001		<.001		.19	

^a^Source: internal administrative member records.

^b^Source: electronic health record (EHR) data.

^c^Source: claims data.

^d^SDI: social determinants index.

^e^AUC: area under the curve.

^f^Values based on a defined threshold of 0.16.

^g^PPV: positive predictive value.

^h^NPV: negative predictive value.

^i^AP: average precision.

**Table 3:** Ranked importance of top features in the final model (M3; 907 total inputs).

Feature category and M3 features		M3 ranked importance^a^
**Demographics (2 inputs)**		
	Age^b^	1
	Gender^b^	2
**Diagnoses (233 inputs)**		
	Chronic respiratory disease	3
	Kidney disease^b^	8
	Total patient diagnoses	17
	Dementia	18
**Medical utilization (265 inputs)**		
	Total patient claims	4
	Average cost of claim	11
	Total CT^c^ scans	13
	Time since last outpatient visit	15
**Pharmacy utilization (198 inputs)**		
	Antihyperlipidemics	5
	Furosemide	7
	Anti-inflammatory analgesics	9
	Beta blockers	14
	Antidepressants	16
	Diuretics	19
**Laboratory utilization (201 inputs) **		
	Systemic and topical nasal agents	20
	Lipid panel lab test	6
**Social determinants index (SDI) score (8 inputs)**		
	Food access	10
	Economy	12

^a^Ranked importance based on positive Shapley Additive Explanations value of features.

^b^M1 feature.

^c^CT: computed tomography.

## Discussion

### Principal Findings

In the past, provider groups and physicians have relied on manual checking of patient records to prescribe palliative care for patients. Today, palliative care teams are increasingly using enhanced decision tools, such as ML approaches, for expedient care delivery. Our palliative care ML model aims to provide a more objective, automated way to identify patients in Medicare Advantage who could most benefit from palliative services, ensuring appropriate clinical resource allocation to the patients with the highest need. The health plan’s goal is to optimize the patient’s quality of life outcomes and incorporate all aspects of palliative care, including care coordination, polypharmacy, symptom management, advanced care plans, as well as spiritual and psychosocial assessments. In this sense, identifying patients who can benefit from a palliative care intervention takes a whole-person health approach to chronic health management and end of life care; the focus is not solely on a transition to hospice. In practice, the model could be deployed within case management, home health, or direct-to-provider programs.

Earlier ML studies of community-dwelling older Medicare beneficiaries have attempted to refine the predictive capabilities of various ML model configurations. However, few have reported outcomes of their specific model feature inputs [25-29]. Understanding important features contributing to mortality prediction algorithms can highlight differences in outcomes between models based on the population studied, ML model approach, and type of data analyzed. Increased transparency in reporting model feature outcomes may also help inform the criterion validity of existing clinical assessment tools used to evaluate patients for palliative care needs. Furthermore, features capturing the SDOH have also been largely neglected from ML models in previous literature [6,25-29,31-33,36,41]. Our feature impact outcomes show that SDOH (ie, food access and local economy) not only are relevant to the prediction of end of life in the community-dwelling Medicare Advantage population but also may be more influential on the outcome than some archetypal high-risk diagnostic and service utilization indicators of palliative care need that are perhaps more commonly observed in hospital settings (eg, ventilator use, sepsis).

The performance of our baseline gradient-boosted machine model predicting 1-year mortality in Medicare Advantage plan members (aged ≥65 years) improved with the incorporation of patient service utilization, diagnoses, and SDOH features. Having access to and adding the full medical, laboratory, and pharmacy claims data and SDI measures enhanced our ML approach. The performance of our model is comparable to that of previous ML studies of older community-dwelling Medicare beneficiaries using claims data (see Multimedia Appendix 4). Some of these models have achieved greater accuracy than that in this study, particularly those models using deep learning configurations. For example, the long short-term memory and deep neural network models developed by Guo et al [25] outperformed their random forest model for predicting mortality in outpatients. Although these types of ML models may achieve greater accuracy, the enhanced model complexity and types of data analyzed by deep learning configurations may not be available or necessary in some cases. Patient medical claims are a common and plentiful source of data that can be used to train binary classification ML algorithms for predicting mortality and other health outcomes. In contrast to inputs already defined within discrete data sets, model inputs generated from raw text might also produce more ambiguous feature definitions that could create challenges for feature impact reporting. Classification models using routine, standard data (ie, claims, administrative records) may be a more attractive option for health plans and other organizations that already collect such data with predefined discrete variables to fulfill their business purposes.

### Limitations

Age and gender were the most influential features in our final model. Although these demographic features had substantial impact on the mortality risk outcome, it is unsurprising that age is the most important model feature, as the probability of death increases with age in older individuals. There is also evidence that, for various reasons, men may be likelier to die earlier than women [42]. The importance of age as a predictive variable is documented in the feature reporting of studies on ML mortality models for hospitalized patients [43]. For community-dwelling Medicare Advantage members over 65 years of age, omitting the age or gender inputs may influence the prediction of mortality risk in cases for which the outcome could be better explained by these demographic variables. Race and ethnicity were purposefully excluded from the model. Race and ethnicity are related to certain disease outcomes, but the literature suggests that social determinants may mediate or modify observed racial or ethnic health differences [44]. When predicting mortality, we believe the composite SDI scores provide more information on the regional variation in individual levels of SDOH and potentially less measurement bias compared with patient race or ethnicity [33].

Our model was developed using only data from a nationwide population sample of community-dwelling Medicare Advantage plan members aged 65 years or older, which could constrain the generalizability of study results to other kinds of patient groups and health settings. Although our model was trained based just on the Medicare Advantage population, bidirectional data sharing between US commercial and other government products would allow for other types of health care consumers to benefit from ML tools for early identification of patients for palliative care. Additionally, our ML model was built to be generic and disease-agnostic. The mortality outcome for the year 2018 encompassed all causes of death, and the feature generation period was also randomized with the span of 1 year. Although the model’s applicability to different patient populations and care settings is still unknown, the generic model can be applied to the plan’s Medicare Advantage members across different years.

### Conclusion

ML offers greater precision and sensitivity in predicting patient end of life and potential need for palliative services among community-dwelling older Medicare beneficiaries. In response to a lack of feature reporting in relevant previous research, this study explored the development of a binary classification ML algorithm predicting 1-year mortality among a sample of Medicare Advantage plan members and investigated the mortality model’s features of top importance. We found the most important features included demographics, diagnoses, pharmacy utilization, mean costs, and certain SDOH. The final ML model predicts mortality among Medicare Advantage plan members with a high degree of accuracy and precision using a variety of routinely collected data and can support earlier patient identification for palliative care.

## Appendix

Multimedia Appendix 1 Health plan process for identifying palliative care patients using machine learning.

Multimedia Appendix 2 Social determinants index (SDI) select measures summary.

Multimedia Appendix 3 Model summary and performance comparison (Training Cohort).

Multimedia Appendix 4 Machine learning (ML) models predicting patient mortality for earlier identification for palliative care.

## Abbreviations

AUC: area under the receiver operating characteristic curve
CPT: Current Procedural Terminology
EHR: electronic health record
LightGBM: light gradient-boosted tree model
M1: Model 1
M2: Model 2
M3: Model 3
ML: machine learning
SDI: social determinants index
SDOH: social determinants of health
SHAP: Shapley Additive Explanations

## References

[1] (2021). March 2021 Report to the Congress: Medicare Payment Policy. Medicare Payment Advisory Commission.

[2] May P, Tysinger B, Morrison RS, Jacobson M (2021). Advancing the economics of palliative care: The value to individuals and families, organizations, and society. USC Schaeffer Center for Health Policy & Economics.

[3] Bevins J, Bhulani N, Goksu SY, Sanford NN, Gao A, Ahn C, Paulk ME, Terauchi S, Pruitt SL, Tavakkoli A, Rhodes RL, Kazmi SMA, Beg MS (2021). Early palliative care is associated with reduced emergency department utilization in pancreatic cancer. Am J Clin Oncol.

[4] Cunningham C, Ollendorf D, Travers K (2017). The effectiveness and value of palliative care in the outpatient setting. JAMA Intern Med.

[5] De Jonge KE, Jamshed N, Gilden D, Kubisiak J, Bruce SR, Taler G (2014). Effects of home-based primary care on Medicare costs in high-risk elders. J Am Geriatr Soc.

[6] Zhang B, Wright AA, Huskamp HA, Nilsson ME, Maciejewski ML, Earle CC, Block SD, Maciejewski PK, Prigerson HG (2009). Health care costs in the last week of life: Associations with end-of-life conversations. Arch Intern Med.

[7] Vallabhajosyula S, Prasad A, Dunlay SM, Murphree DH, Ingram C, Mueller PS, Gersh BJ, Holmes DR, Barsness GW (2019). Utilization of palliative care for cardiogenic shock complicating acute myocardial infarction: A 15‐year national perspective on trends, disparities, predictors, and outcomes. JAHA.

[8] Seow H, O'Leary Erin, Perez R, Tanuseputro P (2018). Access to palliative care by disease trajectory: A population-based cohort of Ontario decedents. BMJ Open.

[9] Etkind SN, Bone AE, Gomes B, Lovell N, Evans CJ, Higginson IJ, Murtagh FEM (2017). How many people will need palliative care in 2040? Past trends, future projections and implications for services. BMC Med.

[10] Yen Y, Hu H, Lai Y, Chou Y, Chen C, Ho C (2022). Comparison of intuitive assessment and palliative care screening tool in the early identification of patients needing palliative care. Sci Rep.

[11] Downar J, Wegier P, Tanuseputro P (2019). Early identification of people who would benefit from a palliative approach—Moving from surprise to routine. JAMA Netw Open.

[12] White N, Reid F, Harris A, Harries P, Stone P (2016). A systematic review of predictions of survival in palliative care: How accurate are clinicians and who are the experts?. PLoS One.

[13] Fischer SM, Gozansky WS, Sauaia A, Min S, Kutner JS, Kramer A (2006). A practical tool to identify patients who may benefit from a palliative approach: The CARING criteria. J Pain Symptom Manage.

[14] Pilsworth S, Wat D, Sibley S, Crane J (2018). A service evaluation of the accuracy of the Gold Standard Framework Proactive Indicator Guidance (GSF PIG) in predicting 12 month mortality in patients with a diagnosis of chronic obstructive pulmonary disease (COPD). Eur Respir J.

[15] Gómez-Batiste X, Turrillas P, Tebé C, Calsina-Berna A, Amblàs-Novellas J (2022). NECPAL tool prognostication in advanced chronic illness: A rapid review and expert consensus. BMJ Support Palliat Care.

[16] Wharton T, Manu E, Vitale CA (2015). Enhancing provider knowledge and patient screening for palliative care needs in chronic multimorbid patients receiving home-based primary care. Am J Hosp Palliat Care.

[17] Thoonsen B, Engels Y, van Rijswijk E, Verhagen S, van Weel C, Groot M, Vissers K (2012). Early identification of palliative care patients in general practice: Development of RADboud indicators for PAlliative Care Needs (RADPAC). Br J Gen Pract.

[18] Highet G, Crawford D, Murray SA, Boyd K (2014). Development and evaluation of the Supportive and Palliative Care Indicators Tool (SPICT): A mixed-methods study. BMJ Support Palliat Care.

[19] ElMokhallalati Y, Bradley SH, Chapman E, Ziegler L, Murtagh FE, Johnson MJ, Bennett MI (2020). Identification of patients with potential palliative care needs: A systematic review of screening tools in primary care. Palliat Med.

[20] Walsh RI, Mitchell G, Francis L, van Driel ML (2015). What diagnostic tools exist for the early identification of palliative care patients in general practice? A systematic review. J Palliat Care.

[21] Davies JM, Gao W, Sleeman KE, Lindsey K, Murtagh FE, Teno JM, Deliens L, Wee B, Higginson IJ, Verne J (2016). Using routine data to improve palliative and end of life care. BMJ Support Palliat Care.

[22] Storick V, O'Herlihy A, Abdelhafeez S, Ahmed R, May P (2019). Improving palliative and end-of-life care with machine learning and routine data: A rapid review. HRB Open Res.

[23] Austin SR, Wong Y, Uzzo RG, Beck JR, Egleston BL (2015). Why summary comorbidity measures such as the Charlson Comorbidity Index and Elixhauser Score work. Med Care.

[24] Gagne JJ, Glynn RJ, Avorn J, Levin R, Schneeweiss S (2011). A combined comorbidity score predicted mortality in elderly patients better than existing scores. J Clin Epidemiol.

[25] Guo A, Foraker R, White P, Chivers C, Courtright K, Moore N (2021). Using electronic health records and claims data to identify high-risk patients likely to benefit from palliative care. Am J Manag Care.

[26] Berg GD, Gurley VF (2019). Development and validation of 15-month mortality prediction models: A retrospective observational comparison of machine-learning techniques in a national sample of Medicare recipients. BMJ Open.

[27] Makar M, Ghassemi M, Cutler DM, Obermeyer Z (2015). Short-term mortality prediction for elderly patients using Medicare claims data. IJMLC.

[28] Hamlet KS, Hobgood A, Hamar GB, Dobbs AC, Rula EY, Pope JE (2010). Impact of predictive model-directed end-of-life counseling for Medicare beneficiaries. Am J Manag Care.

[29] Cary MP, Zhuang F, Draelos RL, Pan W, Amarasekara S, Douthit BJ, Kang Y, Colón-Emeric CS (2021). Machine learning algorithms to predict mortality and allocate palliative care for older patients with hip fracture. J Am Med Dir Assoc.

[30] (2022). Feature Impact. DataRobot.

[31] Avati A, Jung K, Harman S, Downing L, Ng A, Shah NH (2018). Improving palliative care with deep learning. BMC Med Inform Decis Mak.

[32] Sahni N, Simon G, Arora R (2018). Development and validation of machine learning models for prediction of 1-year mortality utilizing electronic medical record data available at the end of hospitalization in multicondition patients: A proof-of-concept study. J Gen Intern Med.

[33] Agarwal R, Domenico HJ, Balla SR, Byrne DW, Whisenant JG, Woods MC, Martin BJ, Karlekar MB, Bennett ML (2022). Palliative care exposure relative to predicted risk of six-month mortality in hospitalized adults. J Pain Symptom Manage.

[34] Hilal S, Brayne C (2022). Epidemiologic trends, social determinants, and brain health: The role of life course inequalities. Stroke.

[35] Silva VDL, Cesse EP, de Albuquerque MFPM (2014). Social determinants of death among the elderly: A systematic literature review. Rev Bras Epidemiol.

[36] Shi Y, Wu Z, Zhang S, Xiao H, Zhao Y (2021). Assessing palliative care needs using machine learning approaches.

[37] Luo W, Phung D, Tran T, Gupta S, Rana S, Karmakar C, Shilton A, Yearwood J, Dimitrova N, Ho TB, Venkatesh S, Berk M (2016). Guidelines for developing and reporting machine learning predictive models in biomedical research: A multidisciplinary view. J Med Internet Res.

[38] (2022). Checking Medicare Eligibility. Medicare Learning Network.

[39] (2022). American Community Survey Data via FTP. United States Census Bureau.

[40] Lundberg SM, Lee SI, Guyon I, Von Luxburg U, Bengio S, Wallach H, Fergus R, Vishwanathan S, Garnett R (2017). A unified approach to interpreting model predictions. Advances in Neural Information Processing Systems 30 (NIPS 2017).

[41] Zhang H, Li Y, McConnell W (2021). Predicting potential palliative care beneficiaries for health plans: A generalized machine learning pipeline. J Biomed Inform.

[42] Regan JC, Partridge L (2013). Gender and longevity: Why do men die earlier than women? Comparative and experimental evidence. Best Pract Res Clin Endocrinol Metab.

[43] Park JY, Hsu T, Hu J, Chen C, Hsu W, Lee M, Ho J, Lee C (2022). Predicting sepsis mortality in a population-based national database: Machine learning approach. J Med Internet Res.

[44] Lorch SA, Enlow E (2016). The role of social determinants in explaining racial/ethnic disparities in perinatal outcomes. Pediatr Res.

