# Supplementary material for: Predicting Patient Mortality for Earlier Palliative Care Identification in Medicare Advantage Plans: Features of a Machine Learning Model
Source: JMIR AI. 2023 Feb 20;2:e42253. doi: 10.2196/42253 (PMC11041411; doi:10.2196/42253)
Supplement: Multimedia Appendix 1 [file ai_v2i1e42253_app1.docx]

**Health Plan Process for Identifying Palliative Care Patients Using Machine Learning**


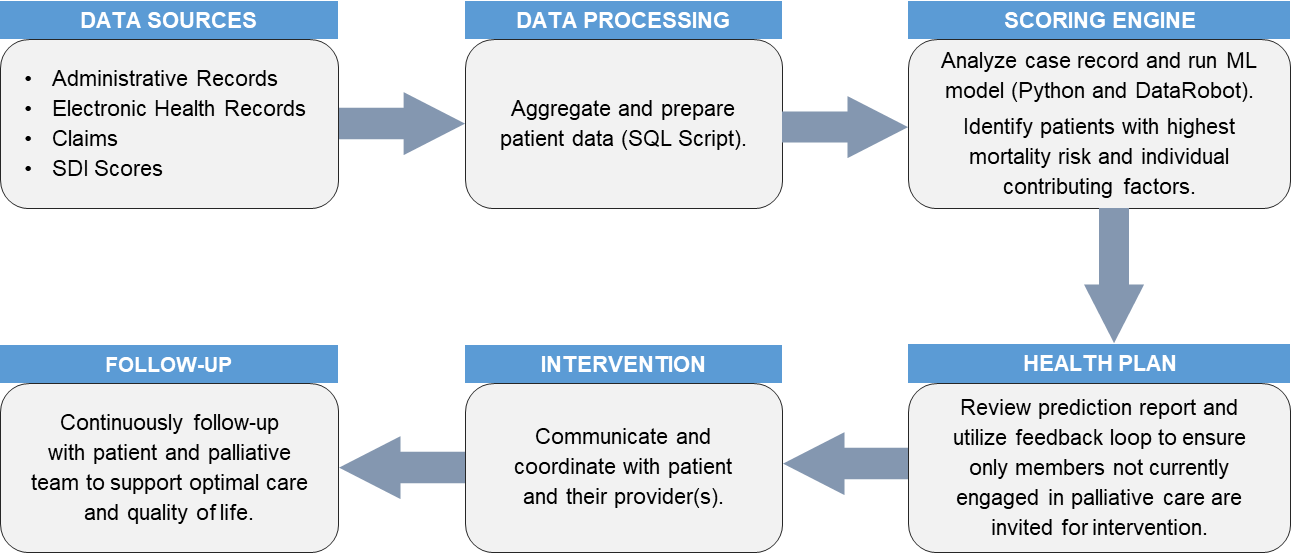


ML: machine learning; SDI: social determinants index.
