# Supplementary material for: Predicting Patient Mortality for Earlier Palliative Care Identification in Medicare Advantage Plans: Features of a Machine Learning Model
Source: JMIR AI. 2023 Feb 20;2:e42253. doi: 10.2196/42253 (PMC11041411; doi:10.2196/42253)
Supplement: Multimedia Appendix 2 [file ai_v2i1e42253_app2.docx]

| **Social Determinants Index (SDI) Select Measures Summary** | | |
| --- | --- | --- |
| *Scoring Domain* | *Examples of Items in SDI Score Calculation* | |
| **Economy** | | - Proportion of households below poverty line |
|  | | - Unemployment rate |
| **Education** | | - Proportion of total population with no high school diploma |
|  | | - Health literacy ratio relative to the national average |
| **Food access** | | - Share of population beyond 1 mile (urban tracts) or 10 miles (rural tracts) from supermarket |
|  | | - Walkability score |
| **Healthcare coverage** | | - Proportion of population 18-34 years of age with no health insurance |
|  | | - Proportion of population 35-64 years of age with no health insurance |
|  | | - Proportion of population >64 years of age with no health insurance |
| **Infrastructure** | | - Proportion of total population who are home owners |
|  | | - Proportion of total housing units that are vacant |
| **Language** | | - Proportion of non-English speakers in total population |
|  | |  |

Note: Total overall SDI scores (unweighted and weighted) of members were also included as model inputs, for a total of eight (8) SDI-related features in the final model.
