# Supplementary material for: Predicting Patient Mortality for Earlier Palliative Care Identification in Medicare Advantage Plans: Features of a Machine Learning Model
Source: JMIR AI. 2023 Feb 20;2:e42253. doi: 10.2196/42253 (PMC11041411; doi:10.2196/42253)
Supplement: Multimedia Appendix 3 [file ai_v2i1e42253_app3.docx]

| **Model Summary and Performance Comparison (Training Cohort)** | | | |
| --- | --- | --- | --- |
| Measure | Model 1 (M1; baseline) | Model 2 (M2) | Model 3 (M3; final) |
| Total model features | 7 | 899 | 907 |
| Model inputs summary | Demographics^a^;  High-risk utilization indicators^b,c^ | Demographics^a^;  High-risk utilization indicators^b,c^;  Medical, lab, and pharmacy utilization^c^ | Demographics^a^;  High-risk utilization indicators^b,c^;  Medical, lab, and pharmacy utilization^c^;  Social determinants index (SDI) scores^a^ |
| *Model Performance* **(Training Cohort)** |  |  |  |
| Area under the curve (95% CI) | 0.737 (0.733-0.741) | 0.831 (0.828-0.834) | 0.836 (0.833-0.839) |
| True positive rate^d^ | 0.110 | 0.320 | 0.338 |
| Positive predictive value^d^ | 0.202 | 0.275 | 0.282 |
| False positive rate^d^ | 0.018 | 0.034 | 0.035 |
| True negative rate^d^ | 0.982 | 0.965 | 0.965 |
| Negative predictive value^d^ | 0.964 | 0.972 | 0.973 |
| False negative rate^d^ | 0.889 | 0.679 | 0.662 |
| Average precision | 0.121 | 0.225 | 0.239 |
| *Performance Comparison* **(Training Cohort)** |  |  |  |
| Null hypothesis | AUC_M1_ = 0.5 | AUC_M2_ – AUC_M1_ = 0.0 | AUC_M3_ – AUC_M2_ = 0.0 |
| *z* statistic | 113.5 | 36.5 | 2.4 |
| *P* value | <.001 | <.001 | .02 |
|  |  |  |  |

^a^Source: internal administrative member records.

^b^Source: electronic health record (EHR) data.

^c^Source: claims data.

^d^Values based against a defined threshold of 0.16.
