# Supplementary material for: Predicting Patient Mortality for Earlier Palliative Care Identification in Medicare Advantage Plans: Features of a Machine Learning Model
Source: JMIR AI. 2023 Feb 20;2:e42253. doi: 10.2196/42253 (PMC11041411; doi:10.2196/42253)
Supplement: Multimedia Appendix 4 [file ai_v2i1e42253_app4.docx]

| **Machine Learning (ML) Models Predicting Patient Mortality for Earlier Identification for Palliative Care** | | | | | | | | | |  | |  |
| --- | --- | --- | --- | --- | --- | --- | --- | --- | --- | --- | --- | --- |
| **Reference** | **ML model purpose** | **Mortality outcome** | **Patients/setting** | **Total n** | **Data source** | **Dates** | **Best ML model** | | **Performance** | |  |  |
| *Older community-dwelling Medicare beneficiaries (aged ≥65 years) using claims data* | | | | | | | | | | |  |  |
| Guo et al (2021) [25] | Predict mortality to identify patients who could benefit from palliative care | Overall status | Medicare beneficiaries (≥65 years) in the Medicare and Medicare Advantage ACOs^a^ of a large integrated health care system operating in 2 states | 59,639 | Claims + EHR^b^ | Jan 2017 - Feb 2020 | | LSTM^c^ | AUC^d^=0.97 | |  |  |
| Berg and Gurley (2019) [26] | Predict mortality to identify patients who could benefit from palliative care | 15-month | Medicare beneficiaries (≥65 years, plus dual eligibles and HMO^e^ enrollees), nationally representative 5% sample in the CMS^f^ Limited Data Set files | 2,714,234 | Claims only | 2014-2015 | LR^g^ | | AUC=0.79 | |  |  |
| Makar et al (2015) [27] | Predict mortality to aid physician and patient decisions regarding EOL^h^ care | 6-month | Medicare beneficiaries (≥65 years), nationally representative 5% sample in fee-for-service | 20,000 | Claims only | 2010 | RF^i^ | | AUC=0.83 | |  |  |
| Hamlet et al (2010) [28] | Predict mortality to identify patients who could benefit from palliative care | 1-year | Medicare beneficiaries (≥65 years) with diabetes and/or heart failure, and with HCC^j^ scores >1.35, in 2 Medicare Health Support pilots (fee-for-service population) | 43,497 | Claims only | 2006-2007 | Neural network | | AUC=0.90 | |  |  |
| *Older hospitalized Medicare beneficiaries (aged ≥65 years) using claims data* | | | | | | | | | | |  |  |
| Cary et al (2021) [29] | Predict mortality to identify patients who could benefit from palliative care | 1-year | Medicare beneficiaries (≥65 years) admitted to Medicare-certified Inpatient Rehabilitation Facilities following hospitalization for hip fracture | 17,140 | Claims + Medicare assessment data | 2015 | MLP^k^ | | AUC=0.75 | |  |  |
| *Adult community-dwelling Medicare beneficiaries (aged ≥18 years) using claims data* | | | | | | | | | | |  |  |
| Zhang et al (2021) [41] | Predict mortality to identify patients who could benefit from palliative care | 1-year | Adult (≥18 years) Medi-Cal and Medicare beneficiaries in a nonprofit regional health care plan in California | 17,197 | Claims only | 2017-2018 | AdaBoost | | AUC=0.73 | |  |  |
| *Hospitalized patients using EHR data* | | | | | | | | | | |  |  |
| Agarwal et al (2022) [33]^l^ | Predict mortality to identify patients who could benefit from palliative care | 6-month | Adult (≥18 years) inpatient admissions at an academic, quaternary health care center in a major metropolitan city in the United States | 176,636 | EHR only (hospital) | 2013-2020 | LR | | AUC=0.87 | |  |  |
| Avati et al (2018) [31]^l^ | Predict mortality to identify patients who could benefit from palliative care | 3-12 months | Adult (≥18 years) and pediatric (<18 years) hospital patients who received care at Stanford Hospital or Lucile Packard Children’s hospital in California | 221,284 | EHR only (hospital) | 1990-2014 | DNN^m^ | | AUC=0.93 | |  |  |
| Sahni et al (2018) [32]^l^ | Predict mortality to aid communication and decision making regarding EOL care | 1-year | Adult (≥18 years) hospital patients within a 6-hospital network in Minnesota | 59,848 | EHR only (hospital) | 2012-2016 | RF | | AUC=0.86 | |  |  |
| Shi et al (2021) [36] | Predict mortality to identify patients who could benefit from palliative care | Overall status | Hospitalization records from adult (≥18 years) and pediatric (<18 years) patients in the State Inpatient Database (SID) of Florida | 1,462,862 | EHR only (hospital) | 2012-2015 | LR | | AUC=0.68 | |  |  |

^a^ACO: accountable care organization,

^b^EHR: electronic health record.

^c^LSTM: long short-term memory.

^d^AUC: area under the curve.

^e^HMO: health maintenance organization.

^f^CMS: Centers for Medicare & Medicaid Services.

^g^LR: logistic regression.

^h^EOL: end of life.

^i^RF: random forest.

^j^HCC: Hierarchical Condition Category.

^k^MLP: multilayer perceptron.

^l^Feature importance reported.

^m^DNN: deep neural network.
